# Genome Characterization of Two *Carrot Virus Y* Isolates from Australia

**DOI:** 10.1128/MRA.00096-20

**Published:** 2020-04-09

**Authors:** Wycliff M. Kinoti, Jane R. Moran, Cherie Gambley, Brendan C. Rodoni, Fiona E. Constable

**Affiliations:** a Agriculture Victoria Research, AgriBio, Bundoora, VIC, Australia; b Department of Agriculture and Fisheries, Nambour, QLD, Australia; c School of Applied Systems Biology, La Trobe University, Bundoora, VIC, Australia; KU Leuven

## Abstract

The near-complete genome sequence of the original *Carrot virus Y* (CarVY) type isolate (CarVY-Vic) collected in 1999 in Victoria, Australia, and a near-complete genome sequence from an isolate collected in 2019 from the same region (CarVY-2-22) were determined following deep sequencing. The two CarVY genome sequences shared 98% nucleotide identity.

## ANNOUNCEMENT

*Carrot virus Y* (CarVY) (family *Potyviridae*, genus *Potyvirus*) is an economically important pathogen of carrots (*Daucus carota* subsp*. sativus*) that causes chlorotic mottle, marginal leaf necrosis or reddening, mild stunting, and increased subdivision of leaflets, giving a “feathery” appearance (1). It has a narrow natural host range and is known to infect only commercially grown and wild *Apiaceae* species (1, 2). CarVY is nonpersistently transmitted by aphids and also is transmitted in seeds (1). CarVY was first reported in Australia in 2002 (2). The type isolate CarVY-Vic (NCBI RefSeq accession number NC_043142.1) is represented only by a 3′-terminal sequence of 1,754 nucleotides (nt) that covers part of the NIb polymerase and the complete coat protein genes and was detected by reverse transcription (RT)-PCR (2).

Tissue from the original carrot sample containing the CarVY-Vic type isolate was preserved in the Agriculture Victoria reference specimen collection, and high-throughput sequencing (HTS) was used to obtain a near-complete CarVY-Vic genome sequence. HTS was also used to obtain a near-complete genome sequence of a second carrot isolate, CarVY-2-22, which was collected in May 2019 from a symptomatic plant from the same region in Victoria, Australia. Total nucleic acids were extracted from the two samples using the QIAxtractor (Qiagen) as described previously (3). A transcriptome sequencing (RNA-Seq) library was prepared using the TruSeq stranded total RNA sample preparation kit with Ribo-Zero Plant (Illumina, San Diego, CA) as described previously (4). The size distribution and concentration of the amplicon libraries were determined using the 2200 TapeStation system (Agilent Technologies) and a Qubit 2.0 fluorometer (Invitrogen), respectively, and the resulting quantification values were used to pool the libraries. The pooled library was diluted, denatured, and sequenced using Illumina HiSeq technology, with a paired-end read length of 2 × 151 bp.

Totals of 16,831,182 and 15,960,819 HTS raw reads were obtained from isolates CarVY-Vic and CarVY-2-22, respectively; after quality trimming using Trim Galore (version 0.6.5) (5), these numbers were reduced to 16,712,236 and 15,641,254, respectively. *De novo* assembly using the SPAdes (version 3.13.0) genome assembler (6) with default settings resulted in 3,416 contigs for the CarVY-Vic isolate and 2,827 contigs for the CarVY-2-22 isolate. A BLASTn search of the GenBank database (7) showed a 9,609-nt contig of the CarVY-Vic isolate and a 9,603-nt contig of the CarVY-2-22 isolate, with 100% and 98% nucleotide identity, respectively, with respect to the original 1,754-nt sequence for CarVY-Vic (NCBI RefSeq accession number NC_043142.1). No other virus contigs were detected by BLASTn analysis of contigs from the two samples. The assembled CarVY-Vic and CarVY-2-22 near-complete genome sequences had GC contents of 38.6% and 39.1% and average coverages of 1,698× and 1,931×, respectively. The CarVY-Vic and CarVY-2-22 near-complete genomes shared 98% nucleotide identity, and both isolates had a complete potyvirus polyprotein coding region with a typical 5′ untranslated region (UTR) and a poly(A) tail 3′ UTR (8). The polyproteins of both isolates translated correctly into a single complete polyprotein coding sequence, and the amino acid similarity was 99%. Overlapping primer pairs were designed (available upon request) for RT-PCR confirmation of only the assembled HTS CarVY genome sequences. Sanger sequencing of the amplicons confirmed the genome sequence arrangements for the CarVY-Vic and CarVY-2-22 isolates. This study reports the first near-complete genomes of two CarVY isolates and provides valuable genome data to facilitate further studies of the genetic diversity of CarVY.

### Data availability.

The genome sequences of the CarVY isolates CarVY-Vic and CarVY-2-22 have been deposited in GenBank under accession numbers LC511903 and LC511904, respectively. The CarVY-Vic and CarVY-2-22 raw data were deposited in the SRA under BioSample accession numbers SAMN14089764 and SAMN14089765, respectively, as part of BioProject PRJNA606340.
